# Molecular Characterization of *Clostridium perfringens* Strains Isolated in Italy

**DOI:** 10.3390/toxins12100650

**Published:** 2020-10-08

**Authors:** Katia Forti, Laura Ferroni, Martina Pellegrini, Deborah Cruciani, Antonio De Giuseppe, Silvia Crotti, Paola Papa, Carmen Maresca, Giulio Severi, Maria Luisa Marenzoni, Monica Cagiola

**Affiliations:** 1 Istituto Zooprofilattico Sperimentale dell’Umbria e delle Marche “Togo Rosati” Via G. Salvemini 1, 06126 Perugia, Italy; l.ferroni@izsum.it (L.F.); m.pellegrini@izsum.it (M.P.); d.cruciani@izsum.it (D.C.); s.crotti@izsum.it (S.C.); p.papa@izsum.it (P.P.); c.maresca@izsum.it (C.M.); g.severi@izsum.it (G.S.); m.cagiola@izsum.it (M.C.); 2 Dipartimento di Medicina Veterinaria, Università degli Studi di Perugia, Via S. Costanzo 4, 06126 Perugia, Italy; marialuisa.marenzoni@unipg.it

**Keywords:** *Clostridium perfringens*, genotyping, toxinotypes, epidemiology, *consensus* and *atypical cpb2*, *cpe*, enterotoxin, clostridial disease, animal sources, food, vaccine

## Abstract

*Clostridium (C.) perfringens* is the causative agent of several diseases and enteric infections in animals and humans. The pathogenicity of the bacterium is largely mediated by the production of a wide range of toxins. Individual *C. perfringens* strains produce only subsets of this toxin repertoire, which permits the classification in seven toxinotypes (A–G). In addition, a variety of minor toxins further characterizes the single strains. The aim of this work was to evaluate, using Polymerase Chain Reaction (PCR) assays, the diversity of 632 *C. perfringens* strains isolated in Italy over 15 years. The genotyped strains were analyzed to determine the presence of major and minor toxins (*cpe*, *consensus,* and *atypical cpb2*), their geographical origins, and the source of isolation (animal species or food). Our study shows that toxinotype A had the greatest representation (93%) and correlated mainly with *consensus cpb2* in a variety of animal species, as well as with *atypical cpb2* in the five food samples. Type D, associated with *cpe* and *atypical cpb2* minor toxins, was identified in 3% of the cases, and type F was identified in 2.5%. Seven type C isolates (1.1%) were detected in cattle, whereas the only type B *atypical cpb2* isolated in Italy was detected in a goat, and one type E *cpe+atypical cpb2* was detected in a sheep. Type G was not detected.

## 1. Introduction

*Clostridium (C.) perfringens* is a saprophyte, anaerobe bacterium with an ubiquitous environmental distribution in soil, sewage, food, feces, and the normal intestinal microbiota of animals, including humans. However, this rapidly growing, widely dispersed, opportunistic, and Gram-positive bacterium becomes, under certain circumstances, one of the most common pathogens that causes several enteric diseases in both in animals and humans, including histotoxic and enteric infections, food poisoning, non-food diarrhea, and enterocolitis [1,2].

The virulence of *C. perfringens* is dependent on the production of at least 20 different toxins and extracellular enzymes [3]. Toxin production, which varies significantly among *C. perfringens* strains, is the basis for a classification system of the *C. perfringens* strains that, has been recently revised to include seven toxinotypes (A, B, C, D, E, F, G), based upon the presence of genes encoding for CPA, CPB, ETX, and ITX toxins, as well as the recently included enterotoxin CPE and necrotic enteritis B-like toxin (NetB) [4].

In addition, individual strains of each toxinotype can produce one or more non-typing toxins, such as CPB2, PFO, NetF, BEC and others. No single strain is known to produce the entire panoply of toxins [5].

The gene encoding CPA toxin (*cpa* o *plc*) is the only localized in a stable region of the chromosome and so this toxin is produced by all *C. perfringens* strains. On the contrary, genes encoding the other typing toxins are encoded on large plasmids; therefore, the *C. perfringens* toxinotyping scheme is fundamentally plasmid-based. In fact, the *C. perfringens* type A that produces only one major toxin CPA represents the basic toxinotype for this species, which upon acquisition of a plasmid encoding for other specific toxins (CPB, ETX, ITX, CPE, NetB) yields another distinct toxinotype (Table A1) [4,6]. Type B encodes the CPA, CPB, and ETX toxins, and type C only encodes the CPA and CPB toxins. Type D isolates produce CPA and ETX. Type E encodes CPA and ITX, which is an intracellular binary toxin (*iap-ibp* genes, globally called *itx*). The new toxinotype F represents the formerly known CPE-positive strains of *C. perfringens* type A and encodes the CPA and CPE toxins upon sporulation. In this case, CPE is a typing toxin because its association with the *cpa* toxin genes has been demonstrated to be responsible for enterotoxigenic infections in humans [7,8] and animals [9]. The new type G produces the CPA toxin and the NetB toxin and is responsible for necrotic enteritis in poultry [10].

CPA is 43 kDa metallic enzyme that exhibited phospholipase C (PLC) and sphingomyelinase (SMase) activities [11]. CPA is the most studied *C. perfringens* toxin and it is an important immunogenic antigen involved in the pathogenesis of enterotoxaemia, as well as in the induction of necrotic lesions in the calf intestinal loop model [12]. Despite the results, the real role of the CPA toxin in intestinal diseases is controversial and subject to extensive debate from the scientific community.

CPB is a thermolabile, trypsine sensitive toxin and it is biological active only in the presence of trypsin inhibitors [10]. CPB is produced by type B and C of *C. perfringens* and is considered to be responsible for fatal hemorrhagic dysentery in sheep (type B) and the fatal intestinal necrosis (e.g., pig-bel disease) seen in type C infections in several animal species (e.g., piglets) and humans [13].

The ETX toxin is produced as a relatively inactive protoxin of 33 kDa that needs proteolytic activation from intestinal proteases to obtain its full functionality [14]. The 27 kDa mature toxin is produced by type B and D and is a potent pore-forming protein responsible for the neurologic signs caused by type D strains. Type D strain correlated with enterotoxaemia disease in sheep and goats [13] and less with enterotoxaemia in calves. ETX has been listed as potential biological and toxic warfare agent and it needs approaches for specific detection and protection [15,16].

The ITX toxin is an intracellular binary toxin composed of two independent proteins. Type E are the only *C. perfringens* strains that produce the ITX toxin and are the putatively uncommon cause of enterotoxaemia in lambs, calves, and rabbits [17].

The *C. perfringens* enterotoxin CPE can be produced mainly by new defined type F [18], but as non-typing toxin can be associated to type A, C, D, and E strains. CPE is a key toxin that induced tight junction rearrangements or pore-formation, causing food-poisoning and non-foodborne diarrhea. The *cpe* gene can be positioned on either the chromosome or on plasmids, and the expression of the toxin only occurs during sporulation.

NetB is a 33 kDa secreted pore-forming toxin encoded by *netB* gene present on a large plasmid [19]. This novel toxin is the first definitive virulence factor to be identified in avian *C. perfringens* strains capable of causing necrotic enteritis (NE). NetB is expressed by virulent new type G strains and causes necrosis of the small intestine in the chicken in a species-specific way [20].

The minor toxin CPB2 is a pore-forming 28 kDa protein, associated with necrotic-hemorrhagic enteritis in piglets [21] and enterocolitis in foals [22]. The *cpb2* gene is of plasmid localization and has two allelic forms called *consensus* (*cons*) or *atypical* (*aty*). CPB2 toxin positive *C. perfringens* strains are widespread and can be isolated from various wild and domestic animals and humans [23], but also from food, soil, and sludge [24]. The exact role of the toxin is not clear but may act in synergy with other major toxins of *C. perfringens* in the production of necrotic and hemorrhagic enteritis. The recently availability of specific reagents against this toxin will improve the studies to understand the biological functions and the pathogenic mechanisms of the CPB2 toxin [25,26].

NetF and BEC are two recently described interesting toxins that are associated to horse and dog diseases [27], as well as possible acute gastroenteritis in humans [28]. These two toxins are candidates for inclusion in a future expansion of the classification scheme and objects of critical experiments [4].

The virulence of different *C. perfringens* isolates, rather than being a function of a single toxin, is considered a multifactorial trait, with different determinants contributing to adaptation of the organism to its niche and to producing the pathological picture [29].

However, the typing of *C. perfringens* strains and conventional classification into seven toxinotypes is important for epidemiology and diagnosis and to differentiate the strains involved in enteric infection because certain toxins are associated with specific hosts and diseases [1].

All the aforementioned species that host the various toxinotypes are relevant in the Italian agricultural and livestock economics, as well as in other countries, although there are territorial and management differences (data from National Livestock Registry, 31 December 2019). Clostridial diseases are a constant threat for the livestock industry globally, responsible for significant economic losses, extensive morbidity, and high lethality rate. Clostridiosis are difficult to eradicate because spores can remain inert for decades in soil and for high levels of animal handling. Due to the rapid and fatal outcomes of this infection, curative treatments are difficult to obtain and there is no immunological cross-protection between the various toxinotypes. Identification of the exact pathogenic toxinotypes circulating in outbreaks is relevant for determining the specific vaccination, i.e., the most effective way to control this disease [30].

The Istituto Zooprofilattico Sperimentale Umbria and Marche “Togo Rosati” (IZSUM) is a Public Health center which works in the Italian territory and especially in its regions of competence (Umbria region and Marche region) to preserve health in terms of food safety and animal welfare, and to support production activities in the agro-food sector by diagnostic activity, food control, and vaccine production. Since 1994, the IZSUM, behind the authorization of the Ministry of Health, has been producing autogenous vaccines (AVs) and autovaccines that are a valid instrument to support veterinarians for both preventive and curative interventions, as well as to give an answer at specific epidemiological situations [31]. AVs are defined as extemporaneous preparations with immunogenic action obtained from a bacterial culture isolated from an infected animal and then used to immunize the animals of the same farm, against further spread and progression of the infection, having essentially a preventive function. AVs represent a valuable contribute in certain situations where suitable commercial vaccines are not available (e.g., clostridial vaccine do not contain the new toxins CPE, netB, the *cons* and *aty* CPB2).

The aim of this study was to molecularly characterize *C. perfringens* isolates collected during 15 years of institutional activities performed by IZSUM. The investigation was carried out using simplex PCR assays to identify the *aty* variant of *cpb2* gene and the *netB* gene, respectively [4,26], as well a new multiplex PCR developed and optimized for this study to identify the *cpa*, *cpb*, *etx*, *itx*, *cpe,* and *cons cpb2* genes. The collection of strains was analyzed with respect to toxinotype, presence of genes coding for minor toxins, geographical origin, and source of isolation. The results provide valuable tools in the development of specific vaccines that setup measures to control clostridial diseases.

## 2. Results

### 2.1. Multiplex PCR of C. perfringens

**Figure 1:**
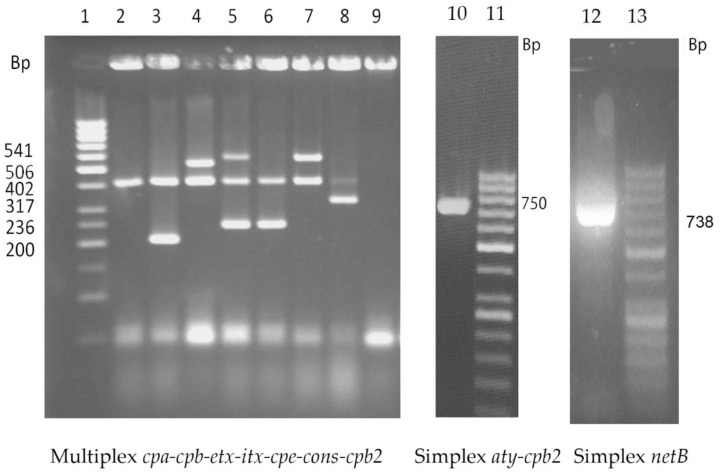
Fragment patterns by multiplex PCR assays of representative reference strains of *Clostridium perfringens*. Multiplex PCR: 50 bp ladder are reported on the left. Lines 1–8: *Clostridium perfringens* strains: ATCC 13124 (type A), KF190/06 (type A-*consensus* CPB2), ATCC 12917 (type F old A-CPE), NCTC 6121 (type B), NCTC 3180 (type C), NCTC 8346 (type D), NTCT 8084 (type E). Line 9: *Clostridium sordellii* ATCC 9714 (negative control). Simplex PCRs Lane 10: FDP82/04 (type A-*atypical* CPB2), Lane 11: 50 bp ladder, Lane 12: strain LBV/18 (type G-*netB*), Lane13: 50 bp ladder.

The multiplex PCR assay presented a high level of sensitivity when performed with the different *C. perfringens* strains. Indeed, defined and predicted bands for each target toxin gene were clearly amplified (Figure 1). PCR products were checked using single simplex PCRs. The specificity of the reaction was confirmed by the absence of amplicons in *C. sordellii* and *C. septicum* strains, which were used as negative controls.

### 2.2. Analysis of Toxinotypes and Prevalence of Minor Toxin Genes

The 632 strains of *C. perfringens* analyzed were classified based on toxinotypes (A to G) and the presence of further non-typing toxin genes coding for *cons* and *aty cpb2* allelic forms, the *cpe* and the two associated genes *cpb2+cpe*. Moreover, the data obtained were also considered according to their geographical origins, as summarized in the Table 1.

Isolates were assigned mainly to type A (588/632, 93%), whereas the other types (B, C, D E, and F) were very limited. No *C. perfringens* type G was isolated.

The majority of the *C*. *perfringens* isolates collected were from the Umbria region (372/632), whereas 219 samples were from other Italian territories (extra-Umbria), and for the remaining 41 isolates, the geographic derivation was unknown (Table 1). Although no statistically significant difference exists among the regions, we can only note that the five toxinotype F in the Umbria region were close and localized in the same district.

The only one isolate assigned to type E was from Umbria, whereas the only type B isolate was from the north of Italy (Piemonte Region). 

**Figure A1:**
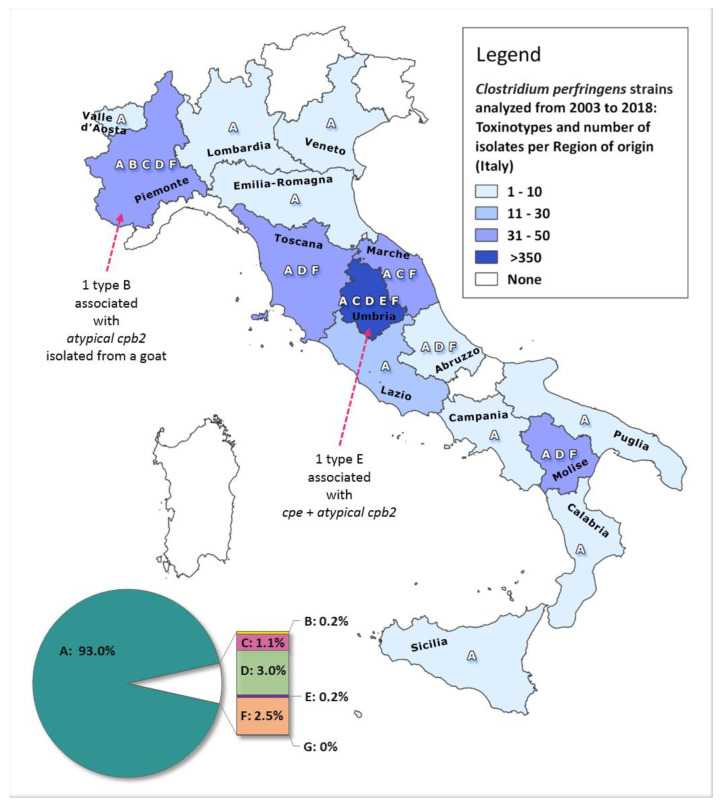
Pie chart of the 632 *C. perfringens* strains by toxinotype. Map of the 591 strains of known origin depicting the number of isolates analyzed and the toxinotypes identified per Italian Region.

The territorial distribution of the 591 isolates of known origin is presented in Figure A1 (Appendix B): the number of isolates analyzed and the toxinotypes identified are reported for each Italian region.

**Figure 2:**
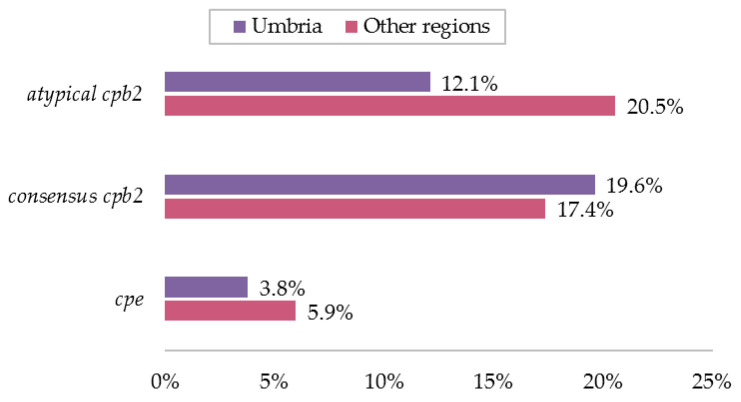
Percentages of minor toxin gene detections, either singularly or in combination, obtained from strains collected from Umbria and the other Italian regions (percentages based on total number of isolates per area).

Figure 2 clearly highlights that the relative prevalence of the two allelic variants *cons-cpb2* and *aty-cpb2* was 19.6% (73/372) and 12.1% (45/372) in Umbria, while it was 17.4% (38/219) and 20.5% (45/219) in the other regions. The statistical analysis conducted on the 591 isolates of known origin revealed no significant differences in the territories for the *cons-cpb2* gene, while the *aty-cpb2* gene was almost twice more likely to be present in the other regions than Umbria (OR 1.88; 95%CI 1.16–3.03; *p*-value = 0.0058).

The presence of the *cpe* gene was about three times lower with respect to the other minor toxin genes in either geographic area (3.8% in Umbria region and 5.9% Other regions) (Figure 2).

An overview of the total number of detections per combination of non-typing gene and toxinotype is provided in Table 2. The *cons-cpb2* gene was the predominant one and it was detected in 19.3% (122/632) of the strains, whereas the allelic variant *aty-cpb2* was detected in 14.9% (94/632). The *cons-cpb2* gene was prevalent in types A and C, while *aty-cpb2* was associated with the other toxinotypes B, D, E, and F.

Beyond Type F, which includes the *cpe* gene by definition and accounted for 2.5% (16/632) of strains, the *cpe* gene was also present as minor toxin coding gene in 2.1% of isolates (12 type D and the only type E). Overall, 4.6% of the strains were enterotoxigenic (i.e., capable of producing the enterotoxin CPE).

Hence, *cpe* (both typing and not) and the two *cpb2* variants were present in 238 isolates out of the total of 632 (37.7%) samples analyzed and they were distributed especially within toxinotypes A, D and F (Table 2).

### 2.3. Association of Toxinotypes and Minor Toxin Genes in Different Animal Species

The results of the toxinotyping for the 632 isolates based on the source of isolation are summarized in Table 3, which provides the ratio of positive isolates with respect to total number of isolations for each matrix, toxinotype, and combination of minor toxin genes.

Among the 632 strains, type A was prevalent (588/632) in all of the different species of domestic and wild animals, birds, and pets, as well as in the foods tested. The only type B strain was derived from a goat and the seven type C strains were derived from cattle, whereas the 19 type D strains were isolated from sheep and goats. The single type E strain was derived from an ovine sample, while the 16 type F strains were of variable origin (three were from cattle, six from sheep, four from goats, and one from a dog).

Of the 236 *C. perfringens* strains isolated from bovine, 226 were type A, and these were associated with both of the *cpb2* allelic variants with a slight prevalence for the *cons* one (16.4% vs. 13.3%).

In total, 184 *C. perfringens* strains were derived from sheep and these were classified as type A (162/184), type D (15/184), type E (1/184), and type F (6/184), respectively. All minor toxins considered in this study (*cpe* and the two *cpb2* variants) were associated with ovine specimens. Moreover, *cons-cpb2* (37/184) was prevalent with respect to the *aty* variant (31/184) and the minor toxin gene *cpe* was present both as a single toxin (9/184) and in combination with *aty-cpb2* (4/184).

The genotypes detected in the caprine isolates (54/632) were of type A (45/54), B (1/54), D (4/54), and F (4/54). Considering the minor toxins, *cons-cpb2* was more frequently represented (13/54) with respect to *aty-cpb2* (9/54), but in this species, a single rare type B strain was found to be associated with the *aty* variant of the *cpb2* toxin gene. The *cpe* gene in caprine specimens was typified (4/54) and so it was only detected in association with the *cpa* gene in the type F (7.4%).

**Figure 3:**
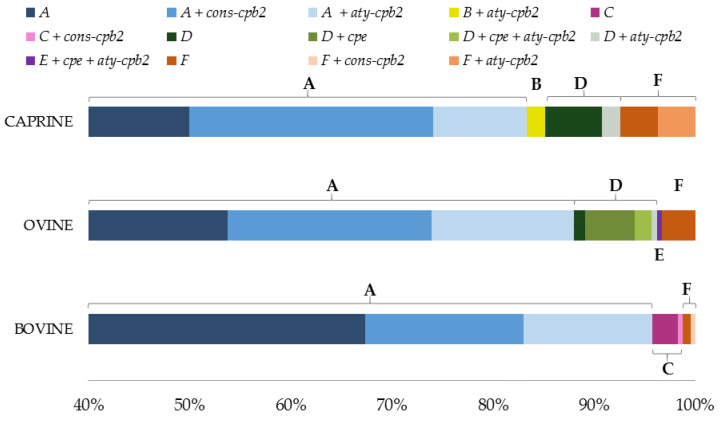
Percentage distribution of toxinotypes and minor toxins genes observed in the most considerable animal species.

The toxinotypes distribution and the minor toxins genes were analyzed in bovine, ovine, and caprine species (the most represented animal species). The results are reported in Figure 3.

The two allelic forms of the non-typing *cpb2* toxin were equally present in rabbits, dogs, and alpacas. In hares and pigs *cons-cpb2* was prevalent, whereas the five strains isolated from food were all of type A and all presented the *aty* allelic variant of *cpb2.* The *C. perfringens* isolates from rabbits and hares were all typed A, and the *cons-cpb2* gene was present in approximately 20% of isolates. However, we found that both *cpb2* allelic variants were equally distributed in rabbits (6/27), but the *cons-cpb2* was mainly specific to the hare species (from a total of 14 type A isolates, 10 were associated with the *cons-cpb2* gene and only two isolates were associated with the *aty* allelic variant).

Overall, the *cons-cpb2* allelic variant was more prevalent in type A strains from all matrices, while the *aty-cpb2* variant showed a major presence in the other toxinotypes. In fact, *aty-cpb2* was present in 2% (5/238) of ovine and caprine type D isolates, in 2/54 of caprine type F isolates, in a quarter of the feline type A isolates, and apparently in one-fifth of the avian isolates, despite the low number of samples collected for this species.

## 3. Discussion

Vaccines are well known strategies for the prevention and control of infectious diseases in animal populations [30]. Their development could be a promising alternative to reduce the use of antibiotics in food-producing animals. *C. perfringens* is normally present in the intestines of animals and humans, but sudden changes in diet, stress temperature, infestation with coccidia, and worms could induce bacterial proliferation and toxin production [32]. Enteric diseases caused by *C. perfringens* are often rapidly fatal, with little time for treatment and important economic impact. The vaccines should provide adequate protective immunity against all the *C. perfringens* toxinotypes circulating in a certain area and limiting the spread of this bacterium at the territorial level. The IZSUM is a benchmark on the regional territories of competence in matters of supervision and control of animal health and production activities in the agro-food sector; moreover, it has the authorization for the production of AVs. These biological preparations are made from the specific bacteria strains isolated from the same flock in which the vaccine is to be use. The diagnostic activity pays a fundamental role and represents the critical point for the AVs production. The use of AVs is only allowed if no licensed vaccines are available, or if the licensed vaccines are relatively ineffective, or if they do not cover the current pathogen strains, or in outbreaks [33].

In the present study, we developed molecular tools for toxinotyping *C. perfringens* strains collected during 15 years of institutional activities and to determine the presence of major and minor toxin genes in these isolates. The data obtained provided an epidemiological picture of the *C. perfringens* toxinotypes and represented a valuable source to develop vaccines that are specific for a certain outbreak or for a particular territory.

The 632 *C. perfringens* strains were genotyped in seven types, A-G, according to the updated recent classification, and these were subtyped to gain further insight into the presence of the minor toxin genes *cpe* and *cpb2* in the *cons* and *aty* allelic forms.

The new classification scheme was presented and approved in August 2017 after being the subject of discussion among many of the authors for several years. However, it has limits and possibilities for improvement. On the one hand, this classification paves the way for the introduction of novel toxinotypes in the future (containing the *netF* and *bec* genes); on the other hand, it lacks of discrimination for the type A that could be considered redundant since the *cpa* toxin gene is present in all strains of *C. perfringens* and describing a strain as type A is no different to describing it as only *C. perfringens*. Another limit to be considered when interpreting the results of the classification by toxinotypes is that the vast majority of the toxins are located on plasmids and these can be lost during bacteriological isolation and culturing. Accordingly, their absence within samples does not mean that they were never there.

Moreover, the collection of strains in the present work cannot be considered representative of the entire Italian territory nor of the national livestock sector, as its composition was allegedly influenced by the territoriality of the IZSUM and its main fields of activities. Not surprisingly, livestock species like cattle and sheep were the most represented source of isolation, albeit this aspect do not differ much from previous studies, as detailed below. Further, socioeconomic and infrastructural differences among territories and breeding type should be considered as possible factors. Despite the data not being complete or exhaustive, the genotyping of *C. perfringens* isolates provides further insight into the presence and characterization of different toxinotypes circulating in Italy from 2003 to 2018.

**Figure A2:**
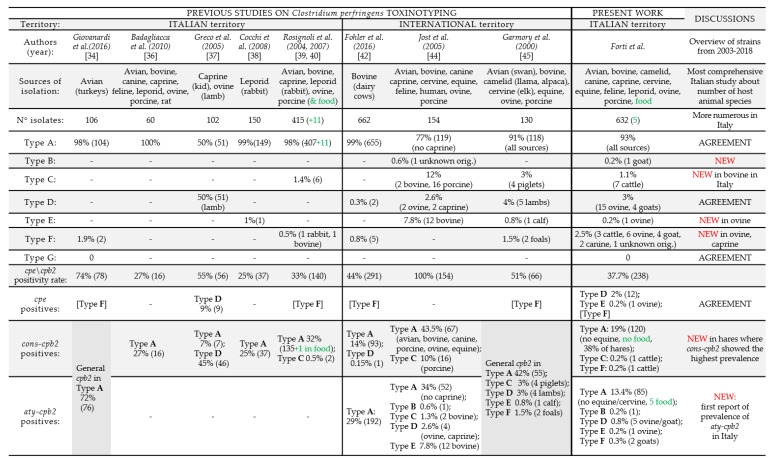
Comparative summary of the most relevant literature (national/international) and the main results of this study; % based on total No. of isolates per study. (*cons-cpb2* = *consensus cpb2*; *aty-cpb2* = *atypical cpb2*).

The obtained results were discussed and compared with existing literature on the molecular characterization of *C. perfringens* at both national and international levels. In order to facilitate and support considerations, a comparative summary of the most relevant scientific publications (Italian and international) and the main results of this study are briefly presented in Figure A2. Each column synthesizes an individual study: the isolates sources, the total number of isolates analyzed, the isolates distribution per toxinotype (percentage and number of isolates per each toxinotype), the total number of isolates, which resulted *cpe*-positive and/or *cpb2*-positive, and the prevalence of these genes per each toxinotype eventually detected (percentage and number of isolates per each toxinotype) are reported for five Italian works and three international studies. The last two columns concern the present study and summarize its primary results and contributions taking into account the previous works. All percentages were computed based on the total number of isolates included in each respective study. Where it was possible, as the information was included in the study, the particular sources of isolation were specified.

The comments on the different topics are discussed point by point below.

### 3.1. Prevalence of Major Toxin Genes in Different Animal Species

Previous studies on the characterization of the types of *C. perfringens* circulating in Italy are limited [34,35,36,37,38,39,40,41]. Recently collected information is available for diseased turkeys [34] and for healthy and ill chickens [35]. Italian studies are restricted to necropsies of a few wild and domestic animal species, namely, diseased rabbits [36,38], cows [41], young lambs and kids [37], and diseased pigs [40]. A more extensive study in a greater number of species and host species was performed by Rosignoli et al. [39,40].

In accordance with other reports [34,35,36,37,38,39,40], the main toxinotype detected in this study was type A, which was associated with many different species, and type D, which was isolated from ovine and caprine species. Greco et al. reported that this is the most predominant cause of enterotoxaemia in very young lambs and kids in southern Italy [37].

Type F (designed as type A-CPE positive) was recently reported in dairy cows [42]. In Italy, its reported presence is limited to turkey isolates [34] and to one rabbit and one of bovine isolates [40]. In accordance with previous literature, in our study, a type F strain was detected in bovine species. In addition, for the first time, it was detected in caprine and ovine species as well as dogs. Moreover, this type circulates significantly in Italy but is less common in Umbria where only 5 strains have been isolated. In this region, all identified types of F were close and localized in the same district. Other authors support the idea that the existence and emergence of some types of bacteria is closely related to geographical characteristics but the number of isolates in our study is too small to support any hypothesis in this regard [43].

The bovine type C and ovine type E strains have not previously been reported in Italy. In fact, the type C strain has only been reported in Italy in pigs [40]. However, in accordance with international surveys, our study confirmed the presence of *C. perfringens* type C in bovine isolates [44]. Moreover, for the first time, we isolated a type E strain in ovine species. *C. perfringens* type E infection has been generally considered a disease with rare occurrences in domestic animals with the exception of a few reports of bovine isolates [44,45,46], diseased rabbits [38], and recently of a neonatal goat with diarrhea [47]. A more detailed discussion of the type E isolate is presented in the next section in association with minor toxins.

Type B infections have been described in the Middle East, Europe, and South Africa with no cases reported in other parts of the world. These cause necro-hemorrhagic enteritis and, more rarely, focal symmetrical necrosis (encephalomalacia) [13]. To the best of the authors’ knowledge, this is also the first investigation to detect a toxinotype B strain in a goat isolate; Jost et al. [44] previously reported this type but did not clarify the specimen.

The *netB* gene is linked to necrotic enteritis in poultry species and the new type G strain was found in a moderate number of healthy human isolates and flocks [48]. Indeed, 15 isolates from 104 chicken samples were *netB* positive (14.4%) [49] and the *netB* gene was found in the feces of clinical isolates in Japan, suggesting transmission between humans and chickens [29].

In our study, no type G strain was detected among the 10 avian isolates. Despite the small number cases, this result is in agreement with an extensive Italian study conducted in 106 *C. perfringens* isolates derived from field strains of diseased turkeys where no *netB* gene was revealed [34]. This finding suggests that, although NetB is a pore-forming toxin with a critical role in starting the damage that initiates necrotic and enteritis, additional unknown virulence factors should be involved and investigated in clinical type G strains isolated from well-defined lesions.

### 3.2. Prevalence of Minor Toxin Genes in Different Animal Species

The subtyping analysis of the *C. perfringens* isolates showed that the *cpe* gene (in its dual role) and the two allelic variants of the *cpb2* gene were associated with 37.7% of the samples. These toxin genes were detected in at least one strain of each toxinotype (A, B, C, D, E, and F).

Moreover, as reported in the results section, the *cons-cpb2* toxin gene was more prevalent than the *aty* variant and the *cpe* gene. This trend was observed in all territories. Comparing the overall Italian percentage data with those of the Umbria specific area, it was found that the *aty-cpb2* gene was less present (12.1%) in the Umbria region than in the rest of the Italian territory (20.5%).

The *cons-cpb2* gene had the highest correlation with the type A strains of bovine, ovine, caprine, leporid, porcine, canine, avian, cervids, alpaca, and camel isolates. It was also found in two isolates: bovine type C and type F, respectively.

Previous Italian studies investigated the *cpe* and *cons-cpb2* genes but not the *aty-cpb2* variant [34,36,37,38,39,40]. The pathogenic role of CPB2 toxin is still debated. Indeed, in some species such as piglets, there seems to be a clear correlation between the lesions and the presence of CBP2; however, in other species, including chickens and humans, this relationship has not been confirmed. The *aty* gene allele encodes for a less toxic CPB2 variant that was not always synthetized due to a frame-shift mutation at position 178 in the *cpb2* gene. The *aty-cpb2* gene is more frequently found in non-porcine isolates of *C. perfringens* [44]. However, the lack of purified CPB2 and specific MAbs has limited the possibility to investigate the pathogenic mechanisms and the biological function of the CPB2 toxin, as well as to develop an efficient immunoenzymatic assay. Recently, Zeng et al. [25] and Serroni et al. [26]. developed these reagents that could be used in further studies to understand the biological activity of CPB2 toxin.

In this study, the *aty-cpb2* gene was observed to have the same prevalence as the *cons-cpb2* variant, and it was especially associated with the type A strains in bovine, ovine, rabbit, avian, dog, and domestic cat isolates. The caprine, alpaca, and avian isolates had lower percentages of the *cons* allelic form. *Aty-cpb2* was present in type D, type F, the unique type B, and type E isolates, and it was the only minor toxin found to be associated with food samples. Moreover, *aty-cpb2* was also associated with the *cpe* gene in three ovine type D isolates and in the unique type E isolate.

Overall, the *cons-cpb2* gene was associated with the isolates of toxinotype A, while the *aty-cpb2* allelic variant was the most widespread on all different toxinotypes. Notably, this overview was valid for all the animal sources with the exception of bovine species which exhibited *cons-cpb2* mostly in type A strains, but also in toxinotypes C and F. On the other hand, ovine and caprine isolates were the only strains which showed the *aty* variant in association with toxinotypes different than type A (Figure 3).

The *cons-cpb2* gene in *C. perfringens* type A had the highest correlation with porcine isolates [40,44], as well as with those isolated from cows [37], rabbits [38], diseased chickens, and turkeys [34,35], whereas it had a variable prevalence in lambs and kids [37].

In agreement with previous works, all the isolates from leporid were molecularly characterized as type A [36,38]. In rabbits both *cpb2* allelic variants were equally found, while in the hares isolates the *cons-cpb2* allelic variant was the prevalent one indicating a possible preference for this species that only future studies with a large number of samples will be able to establish.

The clinical relevance of these non-typing toxins is supported by a recent study on human strains that proposed a further provisional classification for *C. perfringens* strains. The toxinotype H1 was created for the presence of the *cpa* toxins and the minor toxin *cpb2,* and the toxinotype H2 was designed for the presence of the *cpa*, *cpe,* and *cpb2* genes [50]. Regarding the *cpb2* gene, the authors could not discriminate between the two variants, but the molecular tools applied in our study could offer the possibility to differentiate between the two *cpb2* variants and even further characterize the classification into H1 and H2 *cons* and *aty.*

Surprisingly, our study showed that none of the 5 *C. perfringens* deriving from contaminated food presented the *cpe* gene in contrast to that reported in the literature [7,8]. Rosignoli et al. [39,40] reported a unique sample food typed A but associated with the *cons* variant, whereas all our *C. perfringens* isolates were associated with the *aty* allelic variant. These results evidenced for the first time, the association between the type A and the presence of the *aty-cpb2* minor toxin gene in human food poisoning.

The *cpe* gene was found to be especially associated with the *cpa* and *etx* toxin genes (type D) in Umbria, whereas it was only associated with the *cpa* gene (type F) in the other Italian regions. In agreement with the other surveys, the minor toxin *cpe* gene was found to be especially present in type D strain [37] and in the single type E sample associated with the *aty-cpb2* toxin gene [37,45]. A calf isolate type E strain associated with the *cpb2* gene was previously described by Garmory et al. [45], but those authors did not discriminate between the two allelic variants of the *cpb2* gene, so it was not possible to establish a full comparison. Greco et al. [37] associated the *cpe* gene with the *cons-cpb2* gene, whereas in our study, the *cpe* gene was associated with the *aty-cpb2* allelic variant. The percentage of association of the *cpe* gene with the *etx* gene in the toxinotype D (12/19) could be explained by the different localization of the *cpe* locus. In human food poisoning, the *cpe* gene is localized in a variable region of the chromosome carrying the IS*1470* sequence, while in bacteria isolated from human cases of non-food borne gastrointestinal disease or from infected animals, the *cpe* gene is localized on plasmids and is flanked by the IS*1470-*like sequence or the IS*1151* sequence [21,50,51]. The *etx* gene, encoding the ETX toxin, is also linked to the IS*1151* sequence and this suggests that *cpe* and *etx* are located in the same mobile element [6,51].

Regarding the unique type E strain isolated in this study from a sheep in Umbria region and containing both the *cpe* and *aty-cpb2* minor toxin genes, the associated pathological report was compatible with death for hemorrhagic enteritis. *C. perfringens* type E strains are distinguished from other toxinotypes by their simultaneous production of CPA and ITX toxins. CPA toxin is involved in enterotoxemia, but it is likely that a synergism with other factors is needed to cause intestinal necrosis. The lecithinase activity of CPA toxin expressed to the different *C. perfringens* strains could be tested, in egg yolk agar well diffusion turbidity (EYDT) in vitro assay. The culture supernatants of this type E *cpe+aty-cpb2* strain were tested in EYDT in a parallel study conducted to detect the biological activity of rBacCPA250–363H6 compared to that of the native, full length phospholipase C (PLC), which was used as a standard [52]. Preliminary results provided evidence that while the non-toxic rBacCPA250–363H6 protein did not exhibit any lecithinase activity on TSC agar, this type E *cpe+aty-cpb2* strain showed increased CPA activity (in preparation for submission). Other authors reported that the *itx* toxin gene in *C. perfringens* E is present, such as the *etx* gene, on a large plasmid harboring in addition to the *itx* toxin gene. This was also the case for the *cpe* toxin gene and the IS*1151* insertion sequence. Mobilization through conjugation of these large extrachromosomal elements is probably responsible for the variability of *C. perfringens* pathovars seen during biotyping [53]. It is thought that mobile genetic elements carrying various toxin genes insert into plasmid backbones common to many *C. perfringens* strains present in environmental samples and in the feces of healthy individuals. In fact, highly conserved, silent *cpe* gene sequences, located adjacent to the *itx* toxin genes on plasmid DNA, are present in most type E isolates [54]. However, a new type E strain with a plasmid carrying a functional variant of *cpe* gene and *itx* toxin gene was identified in retail meat in Japan, recently [55]. The discovered variant *itx* toxin gene lacks IS elements and carries a functional *cpe* gene. Thus, it is possible that CPE and ITX toxins might act together in pathogenic isolates or they might also be present in healthy or naturally ill people and animals. These finding suggested that the diseases associated to type E require more consideration, especially when taking into account that there are no currently available commercial vaccines containing these toxinotypes.

Considering these results, further investigation should be applied to our strains, particularly to the type D and type E strains associated with an uncharacterized *cpe* locus, as performed in other investigations [28]. Further surveys could be useful to clarify the possible clinical significance, pathology, and to establish whether there is a regional prevalence dominance of type D-*cpe* and to contribute to the knowledge on the evolution of the *cpe* gene itself.

A temporal analysis should be performed to study the distribution of the strains over the years. Moreover, future studies should improve the reliability of the multiplex PCR analysis by including primers capable of detecting the *itx* toxin gene variants, the *netF* and *bec* toxin genes considered for new potential toxinotypes. Additionally, a complete genome sequencing or a MLST analysis could offer valuable insights into the strains circulating in Italy. Further analyses on animals that are not extensively researched (e.g., alpacas, falcons, turtle doves, rabbits, hares, boars, camels, and cats) should also be considered. Whole genome sequencing of these isolates could contribute to good insights into the disease causing mechanisms in these species, providing valuable information for stakeholders.

Finally, *C. perfringens* can be found in the intestine of healthy individuals and the presence of the gene does not implicate that the toxin is produced. Future studies using proteomic and immunological approaches will be directed to establish the real biotypes of the *C. perfringens* strains collected and comparing the genotyping with the toxinotyping will help to establish the real role of the toxins in the disease.

## 4. Conclusions

In this report we provides evidence of the presence of six out of seven *C. perfringens* new toxinotypes in Italy and particularly the diffusion of the *aty-cpb2* toxin gene in this area. Interestingly, a type B strain with the *aty-cpb2* gene was isolated for the first time from a goat isolate and a unique ovine type E strain with associated *cpe+aty-cpb2* genes was isolated from an ovine specimen.

The samples collected in the study were derived mainly from clinical cases of farms distributed throughout Italy. Considering that the characterized strains of *C. perfringens* were mainly the result of severe clinical cases, they represent the main strains that concern the diagnostic activity and therefore represent those that most need to be subjected to control and eradication.

The molecular methodology employed here and the subsequence territorial epidemiological analysis are fundamental for the development of vaccines particularly autogenous vaccines, also known as emergency, herd-specific, or custom made vaccines.

This study currently represents the most comprehensive characterization of Italian isolates in terms of the number of isolates, the number of toxins investigated, and the number of animal species sampled.

The information generated from the study could be used in a surveillance system that makes the data available for all stakeholders. Further links with easily accessible data (for example, quantitative bacteriological parameters, such as the number of unit-forming colonies isolated in clinical cases, the expression of toxins, necropsy results, and farm data) could complete the information and allow analyses to identify possible risk factors and genetic elements of the bacteria that can lead to clostridial diseases, with the aim to better control these infections. Standardization of this workflow, in association with a systematic report, could be useful in the future to allow information to be shared and to build a surveillance system at the national and international levels. Deepened characterization of strains, including those of minor toxins, could be helpful to allow the origins of outbreaks in humans and animals and their possible links to be studied.

## 5. Materials and Methods

### 5.1. Clostridium Perfringens Strain Collection

In total, 632 strains of *C. perfringens* recovered from clinical cases of Clostridiosis in different animal specimens and from contaminated food were collected during 2003–2018 and stored in the Veterinary Diagnostic Laboratory collection (Istituto Zooprofilattico Sperimentale Umbria and Marche “Togo Rosati”, Italy -IZSUM).

The strains were collected from 15 Italian regions, with the most relevant contribution being from Umbria (372 strains), followed by Tuscany (49 strains), Basilicata (45 strains), Marche (40 strains), Piemonte (31 strains), and Lazio (21 strains).

The isolates were obtained from a variety of animal species including bovine (236/632), ovine (184/632), caprine (54/632), leporid (65/632), canine (30/632), porcine (23/632), avian (10/632), feline (8/632), alpaca (8/632), equine (3/632), and corvine (3/632) species. Only five samples were derived from contaminated food.

The isolates came from diagnostic submission for post-mortem examination and were obtained from a wide variety of matrixes as abomasum and rectum content, feces, feed, and food, but also from spleens, livers, bone marrow, kidneys, blood, and intracardiac swabs.

Briefly, the primary isolation from tissues was carried out on 5% sheep blood agar plates after incubation at 37 °C for 24 h. Colonies suspected to have *C. perfringens* were identified using standard Gram stain and biochemical procedures (API20A, bioMérieux Italia Spa, Bagno a Ripoli, FI, Italia). Then, single colonies were subcultured anaerobically on medium thioglycolate broth or Tryptone Glucose Yeast Extract (TGY) broth (3% tryptone, 2% yeast extract, 0.1% glucose, and 0.1% L-cysteine) at 37 °C for 24–48 h. The broth cultures were centrifuged and the pellets were stored at −20 °C and eventually used for genomic DNA extraction. Moreover, all of the isolates were lyophilized in skim milk and stored at +4 °C for collection.

### 5.2. Molecular Characterization

*C. perfringens* genotyping was performed using two protocols. The rapid protocol employed lysed bacterial cells obtained after suspension of 4-5 colonies from overnight cultures on 5% sheep blood agar in 200 µL of distilled water. These were boiled for 10 min at 100 °C, chilled on ice and then centrifuged at 16,000× *g* for 10 min to remove debris. The supernatant was used to perform the PCR tests.

The second protocol used genomic DNA extracted from the bacterial pellet obtained using the QIAamp DNA Mini Kit (QIAGEN, GmbH, Hilden, Germany), in accordance with the manufacturer’s instructions. The bacterial DNA was stored at −20 °C until it was used for PCR reactions.

Template DNA prepared by the direct lysis method was primarily used for isolated strains, but when the results were difficult to interpret or not compliant, extracted genomic DNA was used for confirmation.

Detection of toxin genes was performed using two different types of PCR. A new multiplex assay that was setup and optimized in this study combined primers already reported [56,57]. It was employed for the identification of the *cpa, cpb, etx, iap, cpe*, and the allelic *consensus cpb2* (*cons-cpb2*) genes. A simplex PCR was applied to detect the *atypical cpb2* (*aty-cpb2*) variant of the gene or the *netB* gene of the type G strain [4,26]. Primer sequences and the corresponding lengths of amplicons are shown in Table 4.

Multiplex PCR was performed in a total reaction volume of 25 µL including 2 µL of lysed bacterial suspension or 10–100 ng of purified genomic DNA, 5 µL of 5× PCR buffer, 2 mM of MgCl_2_, 0.2 mM of each of the four dNTPs, 1.25 U of Taq polymerase (Promega Corporation, 2800 Woods Hollow Road, Madison, WI, USA), and 0.8 µM of CPA F-R, 1 µM of CPB F-R, 1 µM of ITX F-R, 1 µM of CPE F-R, 1.4 µM of ETX F-R, 1.4 µM of ETX F-R, and 1.4 µM of *consensus* CPB2 F-R oligonucleotides. Simplex PCR assays for the detection of the *aty-cpb2* minor toxin gene and *netB* gene were carried out as described previously except for 0.5 µM of each specific forward and reverse primers.

The amplification program used for all assays started with an initial denaturation at 94 °C for 5 min, followed by 35 cycles of 1 min at 94 °C, 1 min at 55 °C, 1 min at 72 °C, and a final extension step of 10 min at 72 °C. The PCR products were electrophoresed on 1.5% agarose gel and visualized by MIDORI Green Advance (NIPPON Genetics Europe, Dueren, Germany).

The following *C. perfringens* reference strains were used as controls for the simplex and multiplex PCRs: ATCC 13124 for type A, NCTC 6121 for type B, NCTC 3180 for type C, NCTC 8346 for type D, and NCTC 8084 for type E, ATCC 12917 was used as the positive control for the new type F strain (type A-CPE), whereas strains KF190/06 and FPD 82/04 (IZSUM laboratory collection) were used as the positive controls for the *consensus* and *atypical* CPB2 toxins, respectively. Strain LBV/18 (kindly provided by L. Bano of the IZSVe, Italy) was used as the control for NetB, whereas *C. Sordellii* ATCC 9714 and *C. septicum* ATCC 12464 were used as the negative controls.

### 5.3. Statistical Analysis

The presence of a statistically significant association between specific toxin-coding genes and hypothesized risk factors (geographic origin and source of isolation) was tested using the univariate analysis. Corresponding odds ratios (OR), with 95% confidence interval (95% CI), were determined. Statistical significance was tested using a χ^2^ test, considering a *p* < 0.05 as statistically significant. Statistical analysis was performed with Stata software, version 11.2 (Special Edition, Copyright 2009, Stata Corp LP, College Station, TX, USA). A map representing the territorial distribution of isolates was edited through Qgis 2.18.

## Figures and Tables

**Table 1:** Molecular characterization of *Clostridium perfringens* isolated from 2003 to 2018. The strains were classified based on toxinotype, geographical origin, and presence of further non-typing toxin genes.

No. of Isolates (Intra-Class %)						
Type	Further Toxin Genes	Geographical Origin			Total	Overall Total
		Umbria Region	Other Regions	Unknown		
A	none	237 (63.7)	122 (55.7)	24 (58.5)	383 (60.6)	588 (93.0)
	*cons-cpb2*	72 (19.4)	37 (16.9)	11 (26.8)	120 (19.0)	
	*aty-cpb2*	41 (11.0)	40 (18.2)	4(9.8)	85 (13.4)	
B	*aty-cpb2*	-	1 (0.5)	-	1 (0.2)	1 (0.2)
C	none	4 (1.1)	2 (0.9)	-	6 (0.9)	7 (1.1)
	*cons-cpb2*	1 (0.3)	-	-	1 (0.2)	
D	none	2 (0.5)	3 (1.4)	-	5 (0.8)	19 (3.0)
	*cpe*	6 (1.6)	1 (0.5)	2 (4.9)	9 (1.4)	
	*cpe + aty-cpb2*	2 (0.5)	1 (0.5)	-	3 (0.5)	
	*aty-cpb2*	1 (0.3)	1 (0.5)	-	2 (0.3)	
E	*cpe + aty-cpb2*	1(0.3)	-	-	1 (0.2)	1 (0.2)
F *	none	5 (1.3)	8 (3.7)	-	13 (2.0)	16 (2.5)
	*cons-cpb2*	-	1 (0.5)	-	1 (0.2)	
	*aty-cpb2*	-	2 (0.9)	-	2 (0.3)	
Total		372 (100)	219 (100)	41 (100)	632 (100)	632 (100)

* The presence of the *cpe* gene is considered included in the definition toxinotype F because the CPE toxin is typing in this case; “-“ no isolates.

**Table 2:** Presence of further non-typing genes (either singularly or in combination) per toxinotype.

Type	Typing Genes Combination	Total No. of Isolates per Type	Combination of Non-Typing Genes Detected				
			None	*cons-cpb2*	cpe *	cpe * + aty-cpb2	*aty-cpb2*
A	*cpa-plc*	588	383	120	NA^a^	NA ^a^	85
B	*cpa-plc + cpb + etx*	1	-	-	NA^a^	NA ^a^	1
C	*cpa-plc + cpb*	7	6	1	-	-	-
D	*cpa-plc + etx*	19	5	-	9	3	2
E	*cpa-plc + iap*	1	-	-	-	1	-
F	cpa-plc + cpe *	16	13 (2.06%)	1	NA*	NA*	2
Total		632	407	122	9	4	90
% of 632		100%	64.40%	19.30%	1.42%	0.63%	14.24%

“-“ no isolates; NA = non-applicable; ^a^ gene non-compatible with the relative toxinotype;* *cpe* gene is included in the definition to the toxinotype F because the CPE toxin is typing in this case.

**Table 3:** *Clostridium perfringens* isolates per source of isolation, toxinotype, and combination of non-typing toxin genes detected.

Isolate Source	Total (%)		No. of Positive Isolates per Combination of Non-Typing Toxin Genes / Total No. of Isolate per Toxinotype and Source of Isolation									
			A +	A +	B +	C +	D +	D +	D +	E +	F +	F +
			*cons-cpb2*	*aty-cpb2*	*aty-cpb2*	*cons-cpb2*	*cpe*	*cpe + aty-cpb2*	*aty-cpb2*	*cpe + aty-cpb2*	*cons-cpb2*	*aty-cpb2*
BOVINE:	236	(37.3)	37/226	30/226		1/7					1/3	
cattle	233	(36.9)	36/223	30/223		1/7					1/3	
buffalo	3	(0.5)	1/3	0/3								
OVINE	184	(29.1)	37/162	26/162			9/15	3/15	1/15	1/1	0/6	0/6
CAPRINE:	54	(8.5)	13/45	5/45	1/1				1/4			2/4
goat	53	(8.4)	12/44	5/44	1/1				1/4			2/4
mouflon	1	(0.2)	1/1	0/1								
LEPORID:	65	(10.3)	16/65	8/65								
rabbit	39	(6.2)	6/39	6/39								
hare	26	(4.1)	10/26	2/26								
PORCINE:	23	(3.6)	6/23	1/23								
pig	22	(3.5)	6/22	1/22								
boar	1	(0.1)	0/1	0/1								
CANINE	30	(4.7)	5/28	4/28							0/2	0/2
FELINE	8	(1.3)	0/8	2/8								
CAMELID:	9	(1.4)	4/9	2/9								
alpaca	8	(1.3)	3/8	2/8								
camel	1	(0.1)	1/1	0/1								
EQUINE	3	(0.5)	0/3	0/3								
CERVINE	3	(0.5)	1/3	0/3								
AVIAN	10	(1.6)	1/10	2/10								
FOOD	5	(0.8)	0/5	5/5								
Unknown	2	(0.3)	0/1	0/1							0/1	0/1
Total	632	(100)	120/588	85/588	1/1	1/7	9/19	3/19	2/19	1/1	1	2

Equine: horse; canine: dog; feline: domestic cat; cervine: roe deer, fallow deer; avian: poultry, pheasant, falcon, goose, turtle dove. *cons-cpb2* = *consensus cpb2*; *aty-cpb2* = *atypical cpb2*.

**Table 4:** Oligonucleotide primers used in this study for *Clostridium perfringens* toxin genes detection.

Toxinotypes Detected	Target Gene	PCR	Primer	Sequence (5′--->3′)	AL	[Ref.]
A, B, C, D, E, F, G	*cpa*	M	CPA-F	GTTGATAGCGCAGGACATGTTAAG	402	[56]
			CPA-R	CATGTAGTCATCTGTTCCAGCATC		
B, C	*cpb*	M	CPB-F	ACTATACAGACAGATCATTCAACC	236	[56]
			CPB-R	TTAGGAGCAGTTAGAACTACAGAC		
B, D	*etx*	M	CPETX-F	ACTGCAACTACTACTCATACTGTG	541	[56]
			CPETX-R	CTGGTGCCTTAATAGAAAGACTCC		
E	*iap*	M	CPITX-F	GCGATGAAAAGCCTACACCACTAC	317	[56]
			CPITX-R	GGTATATCCTCCACGCATATAGTC		
F (C, D, E)	*cpe*	M	CPE-F	GGGGAACCCTCAGTAGTTTCA	506	[57]
			CPE-R	ACCAGCTGGATTTGAGTTTAATG		
(A, B, C, D, E, F, G)	*cons-cpb2*	M	CPB2CON-F	CAAGCAATTGGGGGAGTTTA	200	[57]
			CPB2CON-R	GCAGAATCAGGATTTTGACCA		
(A, B, C, D, E, F, G)	*aty-cpb2*	S	CPB2ATY1-25F	AGGAATTCACAAAATGAATACAGTTAAAGCAAATG	750	[26]
			CPB2ATY1-25R	GTGATGATGACCGGTATAACAATAACCCTC		
G	*netB*	S	JRP6656	CTTCTAGTGATACCGCTTCAC	738	[4]
			JRP6655	CGTTATATTCACTTGTTGACGAAAG		

M: Multiplex PCR set up and optimized for this study; S: Simplex PCR; AL: amplicon length (bp); *cons-cpb2* = *consensus cpb2*; *aty-cpb2* = *atypical cpb2*; () in these toxinotypes toxin genes targeted can be optionally present.

## Appendix A. Classification of Toxinotypes

**Table A1:** Molecular typing scheme of *Clostridium perfringens strains* (modified from Rood et al., 2018 [4]).

Toxins:		Typing (Major)						Non-Typing (Minor)		
		CPA-α	CPB-β	ETX-ε	ITX-ι	CPE *	NetB	CPB2-β2	PFO-θ	NetF
*coding gene:*		*cpa-plc*	*cpb*	*etx*	*iap*	*cpe **	*netB*	*cons/aty-cpb2*	*pfo*	*netF*
Toxinotype	A	+						(±)	(±)	(±)
	B	+	+	+				(±)	(±)	(±)
	C	+	+			(±)		(±)	(±)	(±)
	D	+		+		(±)		(±)	(±)	(±)
	E	+			+	(±)		(±)	(±)	(±)
	F	+				+		(±)	(±)	(±)
	G	+					+	(±)	(±)	(±)

CPE *: this toxin is considered minor for the Toxinotype C, D, and E, while it is typing for the Toxinotype F; *cons-cpb2 = consensus cpb2; aty-cpb2 = atypical cpb2*; (±) Toxins in brackets can be optionally present.
